# Synergistic Effects of Salt-Tolerant PGPR and Foliar Silicon on Pak Choi Antioxidant Defense Under Salt Stress

**DOI:** 10.3390/plants14132065

**Published:** 2025-07-06

**Authors:** Jieru Zhao, Qibiao Han, Bingjian Cui, Juan Wang, Chao Hu, Rui Li, Yanyu Lin, Ying Xu, Chuncheng Liu

**Affiliations:** 1 College of Hydraulic Science and Engineering, Yangzhou University, Yangzhou 225009, China; mx120230660@stu.yzu.edu.cn (J.Z.); blesswangj@163.com (J.W.); 2 Institute of Farmland Irrigation, Chinese Academy of Agricultural Sciences, Xinxiang 453002, China; hanqibiao@caas.cn (Q.H.); ayangcbj@126.com (B.C.); huchao@caas.cn (C.H.); lirui01@caas.cn (R.L.); 821012450702@caas.cn (Y.L.); 3 Agriculture Water and Soil Environmental Field Science Research Station of Xinxiang City of Chinese Academy of Agricultural Sciences, Xinxiang 453000, China

**Keywords:** antioxidant enzyme activation, osmolyte accumulation, hormonal regulation, principal component analysis, sustainable saline agriculture

## Abstract

Salinization severely impairs crop growth by inducing oxidative stress and disrupting cellular homeostasis. This study systematically investigates the synergistic effects of salt-tolerant plant-growth-promoting rhizobacteria (ST-PGPR) and foliar silicon fertilizer spraying (FSFS) on antioxidant responses in Pak choi under salt stress. Two-season pot experiments were carried out to evaluate key indicators, including antioxidant enzyme activities (superoxide dismutase: SOD; peroxidase: POD; catalase: CAT), oxidative stress (malondialdehyde: MDA), osmolyte accumulation (proline, soluble protein), and hormones (Jasmonic Acid: JA; Salicylic Acid: SA; Abscisic acid: ABA). The results demonstrate that combining ST-PGPR with FSFS significantly enhances SOD (6.18–2353.85%), POD (3.44–153.29%), and CAT (25.71–319.29%) activities while reducing MDA content (8.12–35.87%). Proline and soluble protein levels increased by 1.56–15.71% and 5.03–188.87%, respectively. Hormonal regulation increased JA, SA, and ABA levels by 1.05–31.81%, 2.09–34.29%, and 3.18–30.09%, respectively. Notably, ST-PGPR treatments at 10^4^ and 10^6^ cfu·mL^−1^, combined with foliar silicon application, consistently ranked highest in overall antioxidant performance across both seasons based on a principal component analysis. These findings provide novel insights into microbial–mineral interactions for sustainable saline agriculture.

## 1. Introduction

Soil salinization has emerged as a critical constraint to global agricultural sustainability, with saline-alkaline soils covering approximately 9.54 × 10^7^ km^2^ worldwide [1]. In China, the saline-alkali lands spans 9.91 × 10^4^ km^2^, leading to reduced crop yields, soil degradation and economic losses [2]. To address this challenge, conventional remediation strategies, including physical methods (e.g., land leveling, soil leaching) [3] and chemical amendments (e.g., gypsum, organic fertilizers) [4], have been widely implemented. However, these approaches face limitations: physical methods are unsuitable for large-scale application as they require high energy inputs, while chemical treatments risk secondary pollution (e.g., heavy metal accumulation) and lack long-term efficiency. In contrast, biological remediation, particularly the utilization of salt-tolerant plant-growth-promoting rhizobacteria (ST-PGPR), offers a sustainable solution by enhancing soil fertility and plant resilience through eco-friendly mechanisms [5,6].

ST-PGPR are rhizospheric microorganisms that thrive in high-salinity environments and directly or indirectly promote plant growth under stress [7]. Their mechanisms [8] primarily include the following: (1) phytohormone synthesis (e.g., indoleacetic acid (IAA)), which stimulates root elongation and nutrient uptake; (2) phosphorus and potassium solubilization, improving soil nutrient availability; and (3) antioxidant enzymes production (e.g., superoxide dismutase, SOD), mitigating oxidative damage caused by salt-induced reactive oxygen species (ROS). For instance, gibberellins synthesized using ST-PGPR increase root hair density, thereby enhancing absorption of nutrient and water required by the plant root system [9] and concurrently improving plant stress resistance and salt tolerance [10]. Furthermore, the ST-PGPR alleviate oxidative damage in crops under salt stress by secreting antioxidant enzymes such as superoxide dismutase (SOD) [11]. Despite these benefits, practical applications of ST-PGPR are hindered by challenges such as microbial instability and susceptibility to the inactivation of single-strain formulations, and inconsistent field performance, especially under fluctuating environmental conditions.

Concurrently, Silicon (Si) fertilization has gained attention for its dual role in alleviating salt stress and improving soil health. Si strengthens plant cell walls through silicification, reducing Na^+^ influx and osmotic stress, while also modulating soil microbial communities [12] by enriching beneficial taxa (e.g., *Actinobacteria* and nitrogen-fixing bacteria), which promotes soil nutrient cycling and crop growth [13]. Foliar Si application furtherly enhances antioxidant capacity by upregulating enzymes like catalase (CAT) and peroxidase (POD), thereby lowering malondialdehyde (MDA) levels—a biomarker for membrane lipid peroxidation [14]. Recent studies have explored the individual effects of ST-PGPR or Si on saline soil remediation; however, the synergistic mechanisms between these two agents in regulating crop antioxidant responses remain poorly understood.

This study aims to bridge this knowledge gap by systematically evaluating the combined effects of ST-PGPR and foliar silicon fertilizer spraying (FSFS) on Pak choi under salt stress. Using a two-season pot experiment, we hypothesize that ST-PGPR and Si act synergistically to (1) enhance antioxidant enzyme activities, (2) reduce oxidative damage, and (3) improve soil moisture-salt dynamics. The findings will advance theoretical frameworks for microbial-Si co-application strategies and provide actionable insights for sustainable saline-alkaline soil management.

## 2. Results

### 2.1. Soil Moisture and Salinity Dynamics

Post-harvest analysis of Pak choi revealed variations in soil moisture content (SMC) under different concentrations of ST-PGPR and FSFS treatments, which are shown in Table 1.

Two-season data reveals distinct trends in SMC across treatments (Table 1). In Season 1, the two-way ANOVA reveals a significant interaction effect between ST-PGPR and FSFS on soil moisture content across both soil layers (0–10 cm and 10–20 cm; F = 5.097, 4.123, *p* < 0.05), whereas neither treatment alone showed significant main effects. In contrast, all factors (ST-PGPR, FSFS, and their interaction) demonstrated non-significant impacts (*p* > 0.05) on soil moisture during Season 2. In both seasons, compared to CK, treatments M_4_, M_6_, and M_8_ exhibited a trend of first increasing and then decreasing in SMC within both 0–10 cm and 10–20 cm soil layers. With Si application, SMC showed a gradually increasing tendency. In Season 1, the combined application (SiM_8_) of ST-PGPR (10^8^ cfu·mL^−1^) and FSFS increased SMC by 36.38% (0–10 cm) and 29.05% (10–20 cm) compared to CK. However, lower ST-PGPR concentrations (SiM_4_, SiM_6_) reduced SMC by 7.65–13.76% (0–10 cm) and 10.49–13.92% (10–20 cm), likely due to microbial competition for water resources. In Season 2, with the addition of silicon fertilizer, SMC showed a gradually decreasing trend. The SMC peaked under SiM_4_ (6.05% increase in 0–10 cm and 11.96% increase in 10–20 cm), indicating seasonal variability in microbial–soil interactions.

A robust positive correlation is observed between soil salt content and electrical conductivity (EC) of a 1:5 soil–water extract. Given the simplicity and practical utility of EC measurement, this parameter is widely used to characterize soil salinity [15]. Post-harvest analysis of Pak choi revealed variations in electrical conductivity (EC) under different concentrations of ST-PGPR and FSFS treatments, which are shown in Table 2.

As shown in Table 2, two-way ANOVA revealed significant main and interactive effects of ST-PGPR and FSFS on soil electrical conductivity (EC; in 0–10 cm, F(ST-PGPR) = 154.639, F(FSFS) = 334.787, F(ST-PGPR × FSFS) = 265.256; in 10–20 cm, F(ST-PGPR) = 150.198, F(FSFS) = 167.817, F(ST-PGPR × FSFS) = 64.046 *p* < 0.01) in Season 1. In contrast, all experimental factors exhibited non-significant impacts (in 0–10 cm, F(ST-PGPR) = 0.748, F(FSFS) = 0.026, F(ST-PGPR × FSFS) = 0.026; in 10–20 cm, F(ST-PGPR) = 1.022, F(FSFS) = 1.864, F(ST-PGPR × FSFS) = 0.890; *p* > 0.05) on EC values across both soil layers during Season 2. Significant differences (*p* < 0.05) were observed among treatments in Season 1, whereas no significant differences (*p* > 0.05) were observed in Season 2. ST-PGPR alone (M_6_: 10^6^ cfu·mL^−1^) reduced EC by 27.63% (0–10 cm) in Season 1 and 14.78% (0–10 cm) in Season 2 (*p* < 0.05), suggesting that the microbial secretion of organic acids mitigated surface salt accumulation [16]. Conversely, FSFS treatments (SiM_4_, SiM_6_) elevated EC by 7.39–9.33% (0–10 cm) and 6.54~10.44% (10–20 cm) in Season 1, possibly due to sodium metasilicate’s ionic contribution; however, FSFS treatments (SiM_4_, SiM_6_, SiM_8_) reduced EC by 6.50~13.90% (0–10 cm) and 0.88~11.38% (10–20 cm) in Season 2.

### 2.2. Antioxidant Enzyme Activities and Oxidative Stress

#### 2.2.1. Superoxide Dismutase (SOD) Activity

**Figure 1:**
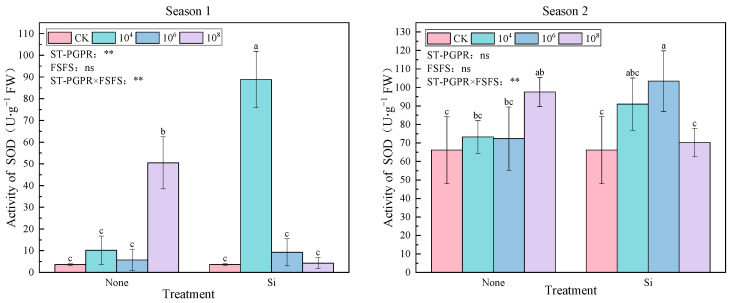
SOD activity under ST-PGPR combined with FSFS. Data are presented as means ±  standard errors (n = 3). Different lowercase letters on the bar indicate significant treatment differences using Duncan’s test (*p*  <  0.05). ** represent a significant difference at the levels of 0.01, respectively; ns represents no significant difference. ST-PGPR: F = 11.088, *p* = 0.001 in Season 1 and F = 1.102, *p* = 0.381 in Season 2; FSFS: F = 3.555, *p* = 0.080 in Season 1 and F = 1.255, *p* = 0.282 in Season 2; ST-PGPR × FSFS: F = 32.574, *p* < 0.001 in Season 1 and F = 7.663, P = 0.0.006 in Season 2.

Figure 1 reveals the SOD activity under salt-tolerant plant-growth-promoting rhizobacteria (ST-PGPR) concentrations combined with foliar silicon fertilizer spraying (FSFS) under salt stress. In Season 1, SiM_4_ (10^4^ cfu·mL^−1^ + FSFS) increased SOD by 2353.85% compared to CK (*p* < 0.05), whereas Season 2 showed maximal activity under SiM_6_ (56.39% increase; *p* < 0.05). In both seasons, compared to CK, all treatments increased SOD activity by 18.46–2353.85% and 6.18–56.39%, respectively. In The SOD activity under treatments without Si application exhibited an initial decline followed by an increase with rising ST-PGPR concentrations. In Season 1, the SOD activity reached the highest under ST-PGPR of 10^4^ cfu·mL^−1^ combining foliar silicon fertilizer spraying. In contrast, the maximum SOD activity was achieved under ST-PGPR at 10^6^ cfu·mL^−1^ and FSFS in Season 2. In the first season, the ANOVA results indicate that ST-PGPR and their interaction had significant effects on SOD activity (F (ST-PGPR) = 11.088, F (ST-PGPR × FSFS) = 32.574, *p* < 0.01), whereas FSFS had no significant effect on SOD activity (F = 3.555 *p* = 0.080). Conversely, ST-PGPR and FSFS had no significant effects on SOD activity (F (ST-PGPR) = 1.102, *p* = 0.381; F (FSFS) = 1.864, *p* = 0.282), whereas their interaction had a significant effect on SOD activity (F = 7.663, *p* < 0.01) in Season 2.

#### 2.2.2. Peroxidase (POD) Activity

**Figure 2:**
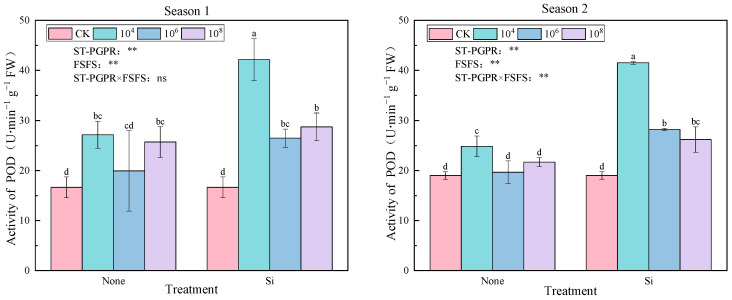
POD activity under ST-PGPR combined with FSFS. Data are presented as means ±  standard errors (n = 3). Different lowercase letters on the bar indicate significant treatment differences using Duncan’s test (*p*  <  0.05). ** represent a significant difference at the levels of 0.01, respectively; ns represents no significant difference. ST-PGPR: F = 10.981, *p* = 0.001 in Season 1 and F = 49.123, *p* < 0.001 in Season 2; FSFS: F = 18.541, *p* = 0.001 in Season 1 and F = 180.066, *p* < 0.001 in Season 2; ST-PGPR × FSFS: F = 3.515, *p* < 0.058 in Season 1 and F = 23.556, *p* < 0.001 in Season 2.

Figure 2 reveals variations in POD activity at different ST-PGPR concentrations combined with FSFS under salt stress. Compared to CK, all treatments elevated POD activity significantly by 19.79–153.29% (Season 1) and 3.44–118.31% (Season 2), with maximal enhancement observed under the ST-PGPR (10^4^ cfu·mL^−1^) + FSFS combination (*p* < 0.05). Notably, foliar silicon application consistently amplified POD activity at equivalent ST-PGPR concentrations (*p* < 0.05), demonstrating a synergistic effect between microbial inoculation and silicon supplementation. In treatments without Si, POD activity exhibited a nonlinear response to ST-PGPR concentrations, characterized by an initial decline at 10^4^–10^6^ cfu·mL^−1^ followed by recovery at 10^8^ cfu·mL^−1^. This pattern suggests a threshold-dependent microbial regulation of POD induction, potentially linked to resource competition or quorum-sensing dynamics. The ANOVA results indicate that independent main effects of ST-PGPR (F = 10.981, *p* < 0.01) and FSFS (F = 18.541, *p* < 0.01) on POD activity were observed, with no significant interaction (F = 3.515, *p* > 0.05) in Season 1. Conversely, both main effects (ST-PGPR: F = 49.123, *p* < 0.01; FSFS: F = 180.066, *p* < 0.01) and their interaction (F = 23.556, *p* < 0.01) significantly influenced POD activity, indicating environmental modulation of microbial-silicon crosstalk.

#### 2.2.3. Catalase (CAT) Activity

**Figure 3:**
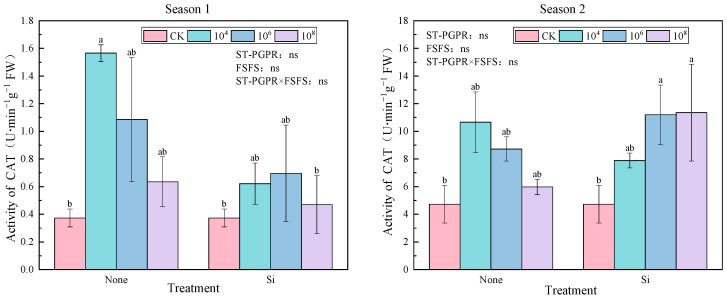
CAT activity under ST-PGPR combined with FSFS. Data are presented as means ±  standard errors (n = 3). Different lowercase letters on the bar indicate significant treatment differences using Duncan’s test (*p*  <  0.05). Ns represents no significant difference. ST-PGPR: F = 2.532, *p* = 0.099 in Season 1 and F = 1.208, *p* = 0.343 in Season 2; FSFS: F = 4.121, *p* = 0.062 in Season 1 and F = 1.298, *p* = 0.274 in Season 2; ST-PGPR × FSFS: F = 0.884, *p* = 0.435 in Season 1 and F = 2.563, *p* = 0.113 in Season 2.

As illustrated in Figure 3, the CAT activity exhibited differential responses to ST-PGPR inoculation and FSFS across two growing seasons. Compared to CK, all treatments increased CAT activity by 25.71–319.286% (Season 1) and 26.33–139.97% (Season 2), suggesting a generalized stress-adaptive response. Without foliar silicon fertilizer spraying application, CAT activity gradually decreased with increasing ST-PGPR concentrations from 10^4^ to 10^8^ cfu·mL^−1^. In Season 1, CAT activity reached a peak under 10^4^ cfu·mL^−1^ ST-PGPR without FSFS. In Season 2, maximum CAT activity shifted to the 10^8^ cfu·mL^−1^ ST-PGPR + FSFS treatment, indicating silicon’s modulatory role in microbial functionality under prolonged stress. A two-way ANOVA demonstrated non-significant main or interactive effects of ST-PGPR and FSFS on catalase (CAT) activity in either growing season (F(ST-PGPR) = 2.532, F(FSFS) = 4.121, F(ST-PGPR × FSFS) = 0.884, *p* > 0.05 in Season 1 and F(ST-PGPR) = 1.208, F(FSFS) = 1.298, F(ST-PGPR × FSFS) = 2.563, *p* > 0.05 in Season 2).

#### 2.2.4. Malondialdehyde (MDA) Content

**Figure 4:**
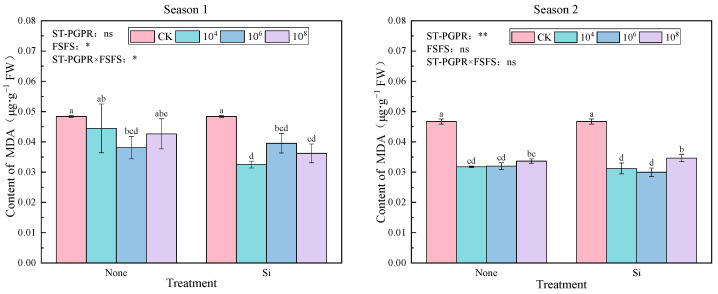
MDA content under ST-PGPR combined with FSFS. Data are presented as means ±  standard errors (n = 3). Different lowercase letters on the bar indicate significant treatment differences using Duncan’s test (*p*  <  0.05). *, ** represent a significant difference at the levels of 0.05 and 0.01, respectively; ns represents no significant difference. ST-PGPR: F = 1.919, *p* = 0.173 in Season 1 and F = 126.334, *p* < 0.001 in Season 2; FSFS: F = 8.042, *p* = 0.013 in Season 1 and F = 1.038, *p* = 0.326 in Season 2; ST-PGPR × FSFS: F = 3.825, *p* = 0.047 in Season 1 and F = 2.645, *p* = 0.106 in Season 2.

As depicted in Figure 4, compared to CK, all treatments reduced MDA content by 8.12–32.81% (Season 1) and 25.86–35.87% (Season 2), with maximal suppression observed at 10^4^ cfu·mL^−1^ ST-PGPR + FSFS in Season 1 and 10^6^ cfu·mL^−1^ ST-PGPR + FSFS in Season 2. In Season 1, a two-way ANOVA revealed the significant impacts of FSFS (F = 8.042, *p* < 0.05) and its interaction with ST-PGPR (F = 3.825, *p* < 0.05) on malondialdehyde (MDA) content, while ST-PGPR alone exhibited no significant effects (F = 1.919, *p* = 0.173). In contrast, during Season 2, ST-PGPR demonstrated a significant effect on MDA content (F = 126.334, *p* < 0.01), whereas FSFS and their interaction showed non-significant impacts (F(FSFS) = 1.038, *p* = 0.326; F(ST-PGPR × FSFS) = 2.645, *p* = 0.106).

### 2.3. Osmolyte Accumulation and Hormonal Regulation

#### 2.3.1. Proline and Soluble Protein

**Figure 5:**
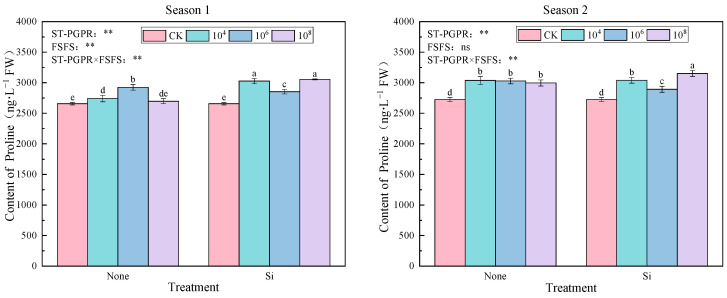
Proline content under ST-PGPR combined with FSFS. Data are presented as means ±  standard errors (n = 3). Different lowercase letters on the bar indicate significant treatment differences using Duncan’s test (*p*  <  0.05). ** represent a significant difference at the levels of 0.01, respectively; ns represents no significant difference. ST-PGPR: F = 9.175, *p* = 0.001 in Season 1 and F = 33.045, *p* < 0.001 in Season 2; FSFS: F = 113.886, *p* < 0.001 in Season 1 and F = 0.131, *p* = 0.722 in Season 2; ST-PGPR × FSFS: F = 55.377, *p* < 0.001 in Season 1 and F = 13.420, *p* = 0.001 in Season 2.

As illustrated in Figure 5, compared to CK, all treatments increased significantly proline content by 1.56–15.02% (Season 1) and 6.19–15.71% (Season 2), with maximal accumulation observed under the 10^8^ cfu·mL^−1^ ST-PGPR + FSFS treatment (*p* < 0.05). At the same ST-PGPR concentration, silicon supplementation exhibited higher proline content than those without Si, indicating a silicon-dependent enhancement of microbial-induced osmolyte production. Intriguingly, proline accumulation in silicon-treated groups displayed a nonlinear response to ST-PGPR concentrations, characterized by an initial decline at 10^4^–10^6^ cfu·mL^−1^ followed by recovery at 10^8^ cfu·mL^−1^. This biphasic pattern suggests a threshold effect, where low-to-moderate microbial concentrations transiently suppress proline biosynthesis until quorum-sensing-mediated stress signaling is activated. ANOVA results indicate that significant main effects of ST-PGPR (F = 9.175, *p* < 0.01), FSFS (F = 113.886, *p* < 0.01), and their interaction (F = 55.377, *p* < 0.01) were observed in Season 1. Conversely, Proline regulation shifted to ST-PGPR dominance (F = 33.045, *p* < 0.01) with a significant interaction effect (F = 13.420, *p* < 0.01), while FSFS main effects became nonsignificant (F = 0.131, *p* = 0.722) in Season 2.

**Figure 6:**
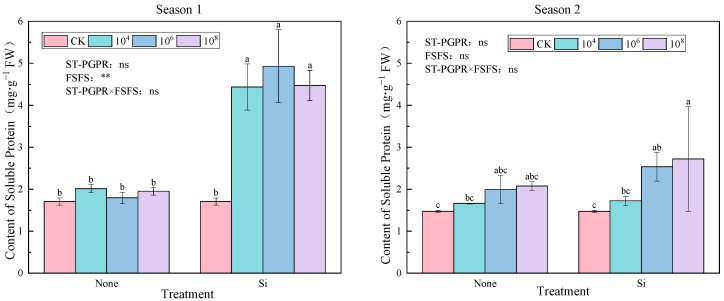
Soluble Protein (SP) content under ST-PGPR combined with FSFS. Data are presented as means ±  standard errors (n = 3). Different lowercase letters on the bar indicate significant treatment differences found with Duncan’s test (*p*  <  0.05). ** represent a significant difference at the levels of 0.01, respectively; ns represents no significant difference. ST-PGPR: F = 0.351, *p* < 0.789 in Season 1 and F = 2.735, *p* = 0.083 in Season 2; FSFS: F = 185.980, *p* < 0.001 in Season 1 and F = 3.021, *p* = 0.104 in Season 2; ST-PGPR × FSFS: F = 1.285, *p* = 0.307 in Season 1 and F = 0.564, *p* = 0.582 in Season 2.

As shown in Figure 6, soluble protein content exhibited distinct seasonal responses to ST-PGPR and FSFS. Compared to CK, all treatments increased soluble protein content by 5.03–188.87%, with a particularly notable enhancement observed when silicon was applied in Season 1. In contrast, Season 2 showed no intertreatment significance (*p* > 0.05) despite substantial SP elevation (12.58–84.51%), suggesting the environmental modulation of protein stability mechanisms. Silicon fertilizer enhanced soluble protein content accumulation across all ST-PGPR concentrations, with maximal values achieved under 10^6^ cfu·mL^−1^ ST-PGPR + FSFS treatment in Season 1 and 10^8^ cfu·mL^−1^ ST-PGPR + FSFS treatment in Season 2. A two-way ANOVA revealed the significant main effect of FSFS (F = 185.980, *p* < 0.01) in Season 1, whereas neither ST-PGPR (F = 0.351, *p* = 0.789) nor its interaction with FSFS (F = 1.285, *p* = 0. 307) showed statistical significance. In contrast, all experimental factors (ST-PGPR, FSFS, and their interaction) demonstrated non-significant impacts (F = 2.735, 3.021, 0.564, *p* > 0.05) on the measured parameters during Season 2.

#### 2.3.2. Phytohormones

**Figure 7:**
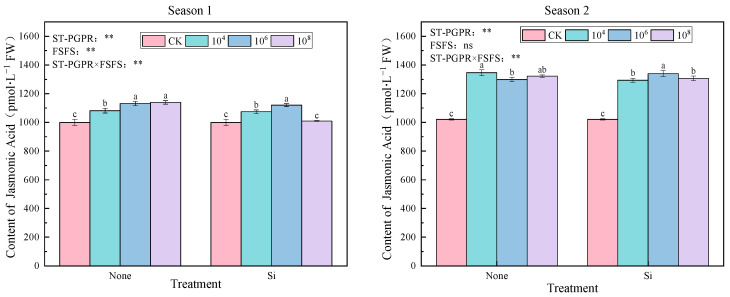
JA content under ST-PGPR combined with FSFS. Data are presented as means ±  standard errors (n = 3). Different lowercase letters on the bar indicate significant treatment differences found with Duncan’s test (*p*  <  0.05). ** represent a significant difference at the levels of 0.01, respectively; ns represents no significant difference. ST-PGPR: F = 70.961, *p* < 0.001 in Season 1 and F = 290.374, *p* < 0.001 in Season 2; FSFS: F = 57.719, *p* < 0.001 in Season 1 and F = 1.487, *p* = 0.243 in Season 2; ST-PGPR × FSFS: F = 36.081, *p* < 0.001 in Season 1 and F = 14.972, *p* < 0.001 in Season 2.

An investigation revealed variations in JA content under ST-PGPR concentrations combined with FSFS under salt stress (Figure 7). Significant treatment effects were observed across both growing seasons (except SiM_8_ treatment in Season 1), with JA levels elevated by 1.05–14.03% (Season 1) and 26.67–31.81% (Season 2) compared to CK. Silicon-amended treatments displayed a biphasic JA response to ST-PGPR density, initial enhancement at 10^4^–10^6^ cfu·mL^−1^ followed by suppression at 10^8^ cfu·mL^−1^, suggesting that microbial quorum-sensing thresholds may regulate JA biosynthesis efficiency. JA content reached the highest at 10^8^ cfu·mL^−1^ ST-PGPR treatment (M_8_) in Season 1 and at 10^4^ cfu·mL^−1^ ST-PGPR treatment (M_4_) in Season 2.

The two-way ANOVA results indicate that there were significant main effects from ST-PGPR (F = 70.961, *p* < 0.01), FSFS (F = 57.719, *p* < 0.01), and their interaction (36.801, *p* < 0.01) in Season 1. However, in Season 2, while ST-PGPR (F = 299.374, *p* < 0.001) and its interaction with FSFS (F = 14.972, *p* < 0.001) continued to show significant influence on JA content, FSFS alone did not exhibit a statistically significant effect (F = 1.487, *p* = 0.243). These findings underscore the complex interplay between biotic and abiotic elicitors in modulating plant defense responses, as mediated by JA signaling.

**Figure 8:**
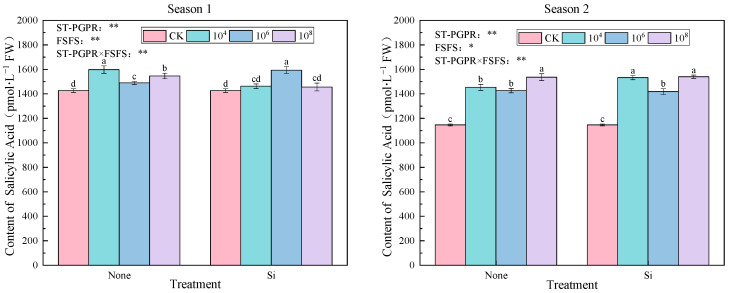
SA content under ST-PGPR combined with FSFS. Data are presented as means ±  standard errors (n = 3). Different lowercase letters on the bar indicate significant treatment differences found with Duncan’s test (*p*  <  0.05). *, ** represent a significant difference at the levels of 0.05 and 0.01, respectively. ST-PGPR: F = 20.988, *p* < 0.001 in Season 1 and F = 237.685, *p* < 0.001 in Season 2; FSFS: F = 12.686, *p* = 0.003 in Season 1 and F = 7.034, *p* = 0.019 in Season 2; ST-PGPR × FSFS: F = 42.086, *p* < 0.001 in Season 1 and F = 8.628, *p* = 0.004 in Season 2.

As illustrated in Figure 8, SA accumulation exhibited concentration-dependent and season-specific modulation under ST-PGPR inoculation and FSFS. Season 1 improved SA contents by 2.09–12.06% compared to CK, especially M_4_, M_8_ SiM_4,_ with significant differences (*p* < 0.05). Significant treatment effects were observed in Season 2 (*p* < 0.05), with SA levels elevated by 23.73–34.29% relative to CK. Non-silicon treatments displayed a distinct biphasic response to ST-PGPR concentrations, initial suppression from 10^4^ to 10^6^ cfu·mL^−1^ followed by recovery at 10^8^ cfu·mL^−1^, suggesting microbial concentration thresholds for SA biosynthesis regulation. Notably, in Season 1, the peak SA concentration was achieved under 10^4^ cfu·mL^−1^ ST-PGPR treatment (M_4_). Conversely, in Season 2, the highest SA concentration was observed under 10^8^ cfu·mL^−1^ ST-PGPR + FSFS treatment (SiM_8_).

The two-way ANOVA results reveal the significant main effects of ST-PGPR (F = 20.998, *p* < 0.01), FSFS (F = 12.686, *p* < 0.01), and their interactive effects (F = 42.086, *p* < 0.01) on SA concentrations in Season 1. However, the main effect of ST-PGPR (F = 237.685, *p* < 0.01), FSFS (F = 7.034, *p* < 0.05) and its interaction with ST-PGPR (F = 8.628, *p* < 0.01) on SA concentrations in Season 2.

**Figure 9:**
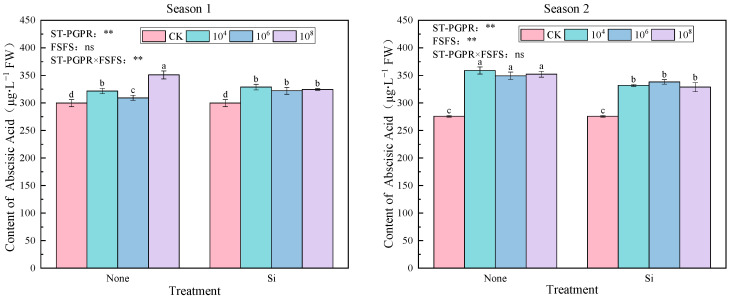
ABA content under ST-PGPR combined with FSFS. Data are presented as means ±  standard errors (n = 3). Different lowercase letters on the bar indicate significant treatment differences found with Duncan’s test (*p*  <  0.05). ** represent a significant difference at the levels of 0.01, respectively; ns represents no significant difference. ST-PGPR: F = 35.815, *p* < 0.001 in Season 1 and F = 153.653, *p* < 0.001 in Season 2; FSFS: F = 0.673, *p* = 0.426 in Season 1 and F = 64.551, *p* < 0.001 in Season 2; ST-PGPR × FSFS: F = 22.660, *p* < 0.001 in Season 1 and F = 3.517, *p* = 0.058 in Season 2.

As shown in Figure 9, ABA accumulation in *Pak choi* exhibited treatment-specific and season-dependent patterns under salt-tolerant plant-growth-promoting rhizobacteria (ST-PGPR) inoculation combined with foliar silicon fertilizer spraying (FSFS). On the whole, significant treatment effects were observed compared to CK in both growing seasons, with ABA contents elevated by 3.18–17.07% (Season 1) and 19.15–30.09% (Season 2). Non-silicon treatments demonstrated a nonlinear response to ST-PGPR concentration: initial decrease at 10^4^–10^6^ cfu·mL^−1^ followed by recovery at 10^8^ cfu·mL^−1^. Seasonal inversion of optimal regimens was evident. In Season 1, the peak ABA concentration was observed under 10^8^ cfu·mL^−1^ ST-PGPR treatment (M_8_). Conversely, the highest ABA content was attained under 10^4^ cfu·mL^−1^ ST-PGPR treatment (M_4_) in Season 2. 

A two-way ANOVA analysis revealed that ST-PGPR (F = 35.815, *p* < 0.01) and interaction effects (F = 22.600, *p* < 0.01) dominated ABA regulation, while FSFS’s main effects were nonsignificant (F = 0.673, *p* > 0.05) in Season 1. However, both ST-PGPR (F = 153.653, *p* < 0.01) and FSFS (F = 64.551, *p* < 0.01) exerted significant main effects, with diminished interaction (F = 3.517, *p* > 0.05) in Season 2.

### 2.4. Comprehensive Evaluation Based on PCA

According to KMO (Kaiser–Meyer–Olkin) and Bartlett’s Test of Sphericity on antioxidant indices of Pak choi across two seasons, KMO values both exceeded 0.5 (0.552 in Season 1 and 0.696 in Season 2), and significance values were less than 0.05, indicating that principal component analysis (PCA) could be used. The PCA was performed to integrate multidimensional antioxidant indices and evaluate the comprehensive effects of salt-tolerant plant-growth-promoting rhizobacteria (ST-PGPR) concentrations and foliar silicon fertilizer spraying (FSFS) across two growing seasons. Three principal components were extracted based on the principle of eigenvalues greater than 1 (Table 3). In Season 1, the first three principal components (PCs) explained 74.84% of total variance (PC1: 36.23%, PC2: 24.93%, PC3: 13.69%). Season 2 increased cumulative variance to 79.07% (PC1: 54.49%, PC2: 13.14%, PC3: 11.43%).

The score formulas for three principal components (Y_1_, Y_2_, Y_3_) are derived by dividing the principal component loading values of each indicator by the square root of the corresponding eigenvalue of the principal component, and then using the coefficients corresponding to each indicator among three principal components, i.e., the eigenvectors, as weights. A comprehensive evaluation model is constructed by linearly weighting and summing the principal component scores and their corresponding weights, calculated based on the proportion of the principal component eigenvalues. The models are as follows: Y = 0.3623Y_1_ + 0.2493Y_2_ + 0.1369Y_3_ (Season 1) and Y = 0.5449Y_1_ + 0.1314Y_2_ + 0.1143Y_3_ (Season 2). The comprehensive scores for each treatment are obtained using this model, and the results are presented in Table 4. The top two comprehensive scores were both SiM_4_ and SiM_6_ across the two seasons, indicating that the overall antioxidant performance of the leaves is optimal at SiM_4_ and SiM_6_.

## 3. Discussion

### 3.1. Synergistic Regulation of Osmolytes by Microbes and Silicon Fertilization

The dynamic balance of osmolytes is a core strategy for plants to maintain cellular homeostasis under salt stress. Soluble proteins, as multifunctional biomacromolecules, stabilize enzyme activity, maintain cell osmotic potential and protect membrane structures under salt stress [17]. AS a vital osmolyte and nutrient, soluble protein enhances cellular water retention, protects biomolecules and membranes, stabilizes enzyme activity, maintains osmotic balance, and participates in stress signaling, serving as a key indicator for stress resistance screening [18]. To maintain osmotic equilibrium and optimal reactive oxygen species (ROS) concentrations under stress conditions, plants synthesize antioxidants and osmoprotectants, such as proline, which is one of the most important osmolytes in response to salt stress [19,20]. Proline, an osmotic protectant, alleviates abiotic stress damage by regulating cellular osmotic potential, scavenging hydroxyl radicals (·OH), and stabilizing protein structures [21]. Previous studies have shown that proline content significantly increases when plants are inoculated with salt-tolerant plant-growth-promoting rhizobacteria (ST-PGPR) [22], and that proline can effectively enhance the salt tolerance and growth of various crops, including olive, tobacco, and wheat seedlings [23,24,25]. In this study, the combined application of ST-PGPR and foliar silicon fertilizer spraying (FSFS) significantly increased the contents of soluble protein (188.87% increase under SiM_4_ treatment in Season 1) and proline (15.71% increase under SiM_8_ treatment in Season 2) (Figure 5 and Figure 6). This aligns with reports of proline accumulation in salt-tolerant species under stress [26] and 20% proline elevation in leaf proline contents when *Eugenia myrtifolia* was treated with 8 dS·m^−1^ NaCl for 30 days [27].

The underlying mechanisms can be interpreted from three aspects: Firstly, at the molecular level, silicon induces the expression of silicon transporters, enhancing the physical barrier of cell wall silica layer to against Na^+^, thereby reducing ion toxicity, which could damage protein structures [12]. Meanwhile, ST-PGPR secrete ACC deaminase, which lowers ethylene levels and alleviates the inhibition of stress-related protein synthesis (e.g., late embryogenesis abundant proteins and heat shock proteins) [8,18]. This mechanism aligns with stabilization of osmoprotective proteins [28]. Secondly, metabolic pathways are activated. The accumulation of proline exhibits a biphasic response: at low ST-PGPR concentrations (10^4^–10^6^ cfu·mL^−1^), silicon upregulates expression of proline synthesis enzyme (P5CS) genes to promote synthesis, while at high concentrations (10^8^ cfu·mL^−1^), ST-PGPR inhibit activity of proline degradation enzyme (ProDH) to reduce degradation [22]. This dose-dependent regulation is highly consistent with the “stress signal threshold” theory, which posits that a specific microbial density is necessary to trigger the synthesis of osmolytes [29]. Thirdly, from an ecological perspective, the seasonal differences in proline content under silicon treatment (15.02% increase under SiM_8_ in Season 1 vs. 15.71% under SiM_8_ in Season 2) suggest that silicon may indirectly stimulate osmolyte accumulation by modulating the rhizosphere microbial community [13].

### 3.2. Effects of ST-PGPR and FSFS on Antioxidant Enzyme Activities

Stress-resistance traits in plants are characterized by their capacity to counteract abiotic stresses (e.g., salinity, drought, extreme temperatures) through integrated physiological, biochemical, and molecular mechanisms. These mechanisms include antioxidant defense systems, osmotic adjustment, stress signal transduction [30]. Specific indicators of stress resistance include enzymes such as superoxide dismutase (SOD), peroxidase (POD), catalase (CAT), and thioredoxin peroxidase, as well as biochemical molecules like hydrogen peroxide (H_2_O_2_), superoxide anion (O_2_^−^), proline, malondialdehyde (MDA), and total antioxidant capacity [31]. Antioxidant enzymes collectively function to catalyze the conversion of reactive oxygen species (ROS) into less toxic or non-toxic compounds, thereby mitigating oxidative stress-induced cellular damage [32].

Superoxide dismutase (SOD) scavenges superoxide anion radicals (O_2_^−^) via dismutation reactions, converting them to hydrogen peroxide (H_2_O_2_) and molecular oxygen (O_2_). This activity is critical for maintaining cellular redox homeostasis [33], thereby underpinning its essential role in the biological antioxidant system. Peroxidase (POD) catalyzes the oxidation of phenolic and amine compounds by hydrogen peroxide (H_2_O_2_), facilitating the decomposition of H_2_O_2_ while also playing a pivotal role in cell wall lignification and scavenges reactive oxygen species (ROS), thereby fulfilling dual functions in the elimination of H_2_O_2_ and detoxifying phenolics/amines [34]. Catalase (CAT), the primary H_2_O_2_-scavenging enzyme, specifically catalyzes the decomposition of H_2_O_2_ into water (H_2_O) and oxygen (O_2_), thereby mitigating oxidative damage from reactive oxygen species (ROS) accumulation [21]. Collectively, this enzyme modulates redox homeostasis and stress resistance in plants under abiotic stress conditions. Salt stress induces the formation of reactive oxygen species (ROS), including superoxide, singlet oxygen, hydroxyl radicals, and hydrogen peroxide, which cause severe damage to cell structures through oxidative stress [35]. ROS generation is linked not only to their intrinsic roles in developmental processes but also to the regulation of morphogenetic processes associated with phytohormones such as cytokinins and auxins [36].

(1) Direct antioxidant defense: Plants counteract oxidative stress through a dynamic balance of the antioxidant enzyme system (superoxide dismutase [SOD], peroxidase [POD], and catalase [CAT]) and lipid peroxidation markers (malondialdehyde [MDA]). SOD acts as the first line of defense against superoxide anions (O_2_^−^), converting them into hydrogen peroxide (H_2_O_2_) to mitigate oxidative stress [37]. CAT decomposes H_2_O_2_ into water (H_2_O) and oxygen (O_2_) to prevent cellular damage [38]. POD also scavenges hydrogen peroxide and strengthens cell wall defenses [39]. These antioxidant enzymes are crucial for eliminating free radicals produced under abiotic stress conditions [40,41]. PGPR application reduces MDA accumulation, electrolyte leakage and membrane damage by enhancing antioxidant enzyme activities and non-enzymatic antioxidants [42].

(2) Hormone-mediated regulation: Many PGPR produces salicylic acid (SA) and strengthen tolerance to both biotic and abiotic stresses by modulating the antioxidant defense system [42]. For example, *B. firmus SW5* eliminates ROS through activation of antioxidant enzymes (e.g., APX, CAT, SOD, POD) under salt stress, accompanied by the upregulation of corresponding genes [43]. Concurrently, PGPR reconfigure phytohormone crosstalk: ABA suppression reduces ROS burst sensitivity, while SA/JA synergy amplifies defense gene transcription [44].

(3) System resistance: MAPK cascades are important signaling pathways regulating ROS homeostasis. For example, the MEKK1-MKK1/MKK2-MPK4 cascade controls scavenging enzyme activities [45]. In inoculated plants subjected to salt stress, gene expression analyses revealed a down-regulation of the ZmNCED gene and an upregulation of the ZmDREB2A and ZmWRKY58 genes, two transcription factors that play a critical role in improving plant salt tolerance [46]. Similar results were obtained in Triticum aestivum inoculated with Arthrobacter protophormiae SA3 and Dietzia natronolimnaea STR1 [47]. Additionally, Ca^2+^ channels interact with kinases, lipid-binding proteins, and ROS metabolic enzymes to form stress-sensing complexes [48].

Previous studies have shown that PGPR inoculation significantly elevates antioxidant enzyme activities under saline conditions. For example, okra plants inoculated with PGPR exhibited significantly higher activities of CAT and SOD compared to non-inoculated plants under saline conditions [49]. Similar results were observed in potato plants inoculated with PGPR under stress conditions [50]. Additionally, IAA-producing bacteria have been shown to promote growth under saline stress via increased plant salt tolerance [51,52]. The endophytic bacterium *Bacillus amyloliquefaciens* RWL-1 strengthened salt tolerance in rice [53]. The antioxidant activity in the leaves of rice seedlings increases with increasing salt concentration, and this activity is significantly intensified following inoculation with salt-tolerant plant-growth-promoting rhizobacteria (ST-PGPR) [22]. PGPR can alleviate salt-induced osmotic stress, restore ion balance, and reinforce the antioxidant defense mechanisms in rice [54]. Plants activate their antioxidant defense systems under stress to effectively scavenge reactive oxygen species (ROS), thereby improving adaptability and survival rates [55,56]. PGPR potentiated salt tolerance by regulating osmotic pressure, increasing antioxidant enzyme activities, and modulating the expression of stress-related genes [57].

In this study, SOD activity was improved under the combined treatment of ST-PGPR and FSFS, consistent with findings that show that SOD activity depends on the intensity of stress signals and the availability of cofactors [33]. Silicon may upregulate the expression of silicon transporters, thereby strengthening the physical barrier of the cell wall silica layer against ROS diffusion and indirectly reducing the generation of O_2_^−^ [58]. Meanwhile, ST-PGPR secrete IAA, which activates the expression of the SOD2 gene, thereby enhancing enzyme activity [9]. This synergistic effect led to a maximum increase in SOD activity of 2353.85% (SiM4 in Season1; Figure 1). Additionally, ACC deaminase secreted by ST-PGPR lowers ethylene levels, alleviating the inhibitory effect of ROS signaling on SOD activity [8].

The responses of POD and CAT were treatment-specific. ST-PGPR combined with silicon significantly promoted POD activity, indicating that the synergistic effect of ST-PGPR and silicon is more conducive to H_2_O_2_ scavenging. This may be achieved through silicon-mediated lignin deposition, which reinforces cell wall defenses [59]. In contrast, ST-PGPR-coupled Si treatments decreased the CAT activity compared to ST-PGPR alone (Except 10^6^–10^8^ cfu·mL^−1^ in Season 2), possibly due to silicon’s role in inhibiting H_2_O_2_ accumulation and reducing the demand for CAT induction [60]. This finding corroborates previous research indicating that silicon reduces H_2_O_2_ accumulation, thereby lowering the need for CAT induction [61]. The differences in CAT activity between different growing seasons may be caused by environmental factors.

Excessive ROS production is associated with plants’ responses to pathogens, injuries, ABA, ozone, and heavy metals [62,63]. Lipid peroxidation, a process where ROS oxidize fatty acids, leads to membrane damage. Malondialdehyde (MDA), a key product of membrane lipid peroxidation, reflects the extent of ROS-induced membrane damage [64]. Elevated MDA levels exacerbate membrane injury, making it a common indicator in studies of plant senescence and stress resistance [65].

In this study, compared to CK, MDA content was reduced under ST-PGPR or coupling silicon treatments, consistent with previous findings that plant inoculation with microbial agents lowers MDA levels under salt stress [66]. Alharbi et al. [67] also found that the application of PGPR and ZnO nanoparticles to wheat improved N and Zn assimilation by enhancing antioxidant enzyme activity and potassium absorption, and reduced the stress marker MDA. The combined application of ST-PGPR and silicon reduced MDA content (Figure 5), and the underlying mechanisms may include the following: (1) silicon deposition, enhancing cell wall mechanical strength to reduce direct ROS attacks on plasma membranes [68]; and (2) ST-PGPR-secreting siderophores to chelate Fe^2+^, inhibiting the Fenton reaction [69] and thereby lowering MDA content.

### 3.3. Interactions Between Hormonal Networks Regulated by Microbes and Silicon

Plant hormones, also known as phytohormones, are small chemicals that play crucial roles in plant growth and development. Among the well-characterized plant hormones, abscisic acid (ABA), ethylene, salicylic acid (SA), and jasmonic acid (JA) are regarded as stress response hormones [70].

Abscisic acid (ABA) is the central regulator of plant abiotic stress responses, orchestrating adaptive mechanisms through stomatal aperture modulation to limit transpirational water loss, the transcriptional activation of antioxidant defense genes, and coordinated induction of osmoprotectant biosynthesis pathways under saline conditions [71]. Salicylic acid (SA) plays a pivotal role in enhancing plant resistance to pathogens and environmental stresses by activating systemic acquired resistance and antioxidant enzyme systems [72]. Jasmonic acid (JA), a lipid-derived signaling molecule, regulates plant defense responses to both biotic and abiotic stresses [73].

ABA is responsible for plant defense against abiotic stresses because environmental conditions such as drought, salinity, cold, heat stress, and wounding are known to trigger an increase in ABA levels [74]. ABA accumulation is positively correlated with stress intensity [31]. In this study, silicon application generally reduced ABA content compared to ST-PGPR alone, likely by enhancing root water absorption efficiency and alleviating cellular osmotic stress signals [68]. This antagonistic effect is supported by previous findings that silicon reduces Na^+^ uptake through a physical barrier, partially substituting for ABA’s role in stomatal regulation [61]. Additionally, ACC deaminase secreted by ST-PGPR reduces ethylene accumulation, indirectly inhibiting NCED gene expression, which is consistent with previous research on the alleviation of ABA over-accumulation by the combined application of silicon and microbes [75]. Verma et al. [76] found that ABA accumulates under osmotic stresses as the expression levels of several ABA biosynthesis genes are upregulated by drought and salt stress. ABA induces the expression of a catalase (CAT1), a scavenger of H_2_O_2_, while simultaneously activating H_2_O_2_ production [77]. A previous study has shown that ABA, ROS, and Ca^2+^ exhibit complex signaling crosstalk to control plant growth responses to salt stress [70]. Zhao et al. [78] found that ABA levels increase rapidly under salt stress. ABA responds to salt stress mainly by regulating the stomatal opening and inducing the expression of resistance genes [79].

SA, JA, and Ethylene play major roles in response to biotic stress conditions as their levels increase with pathogen infection [77]. JA has been shown to positively regulate plant tolerance to salt [80,81]. In this study, JA and SA exhibited synergistic defense effects, with ST-PGPR alone or coupled with silicon significantly increasing the contents of both JA and SA. This may be attributed to the reduction in ethylene levels by ACC deaminase secreted by ST-PGPR, which alleviates inhibitory effect of ethylene on JA synthesis [8]. Additionally, JA activates MYC2 transcription factor to facilitate ABA signaling [82]. In the absence of silicon, ST-PGPR alone increased SA content, likely due to activation of phenylalanine ammonia-lyase (PAL) by ST-PGPR, which raises SA synthesis [83]. This antagonistic relationship was first reported in tomato, where JA-related wound responses were inhibited by aspirin, an acetylsalicylic acid drug [63]. Plants modulate the relative abundance of SA, JA and Ethylene levels, modify the expression of defense-related genes, and coordinate complex interactions between defense signaling pathways to activate an effective defense response [77]. Yu et al. [70] found that SA application not only optimized antioxidant system and the synthesis of osmolytes, but also promoted plants photosynthesis under salt stress. Zheng et al. [84] showed that SA activated the expression of *P5CS*, which is responsible for proline accumulation under salt stress, while reducing H_2_O_2_ concentration.

In *Arabidopsis* seedlings, approximately 10% of the genome is modulated by ABA, with induced and repressed genes equally represented—a proportion two- to six-fold higher than that regulated by other plant hormones [85]. In *B. japonicum*-inoculated roots, upregulation of *RD20*, *CAT3*, and *MDAR2* was observed. Under salt stress, *B. japonicum* additionally reduces expression of *DHAR*, *MSD1*, *MYC2*, and *RD22* in shoots, while downregulating *ADC2*, *ANAC055*, *DHAR*, *GTR1*, *RD20*, *RD29B*, *VSP1*, and *VSP2* in roots [66]. Similarly, CNBG-PGPR-1 reduces salt-induced ROS by activating ROS scavenging genes, primarily involved in glutathione (GSH) metabolism and peroxidase (POD)-related genes, which are closely associated with methionine metabolism [86]. These studies collectively underscore the critical importance of gene expression data and hormone levels in deciphering PGPR-mediated stress resilience. The current study focuses on physiological responses and acknowledges the limitations of not addressing gene expression. Future research will include key antioxidant genes and stress-related genes to rigorously verify the molecular mechanisms involved.

## 4. Materials and Methods

### 4.1. Experimental Site and Soil Preparation

The experiment was conducted in the Agricultural Water and Soil Environmental Field Science Research Station of Xinxiang City of Chinese Academy of Agricultural Sciences (35°19′ N, 113°53′ E, altitude 73.2 m). The average annual temperature was 14.1 °C, the average annual precipitation was 588.8 mm, the average annual evaporation was 2000 mm, the frost-free period was 210 days, and the sunshine duration was 2398.8 h. The tested soil was collected from adjacent farmland, air-dried, ground, and sieved through a 2 mm mesh for subsequent use. Soil properties were determined as follows: pH = 8.49 (soil: water = 1:2.5), electrical conductivity (EC) = 863.98 μS·cm^−1^ (soil:water = 1:5), organic matter = 9.736 g·kg^−1^, field capacity = 21.59%, and total soluble salts = 0.23%. Each pot (top diameter: 24.7 cm; bottom diameter: 29 cm; height: 22.5 cm) was filled with 12 kg of prepared soil.

### 4.2. Experimental Design and Treatments

“Pak choi” (Shanghai Green) was selected as the experimental plant due to its sensitivity to salt stress. Seeds were sown on 9 September 2023, and 9 March 2024, to represent two growing seasons.

A two-factor completely randomized design was applied: salt-tolerant plant-growth-promoting rhizobacteria (ST-PGPR) and foliar silicon fertilizer spraying (FSFS). ST-PGPR included 3 levels: 10^4^, 10^6^, and 10^8^ cfu·mL^−1^, respectively.

A single bacterial strain was isolated from rhizosphere soil of a saline-alkali field through dilution screening, plate streaking, and purification. The strain’s characteristics were systematically evaluated as follows: (1) it exhibited indoleacetic acid (IAA) production, confirmed by a red-colored reaction with Salkowski reagent; (2) it demonstrated phosphate and potassium solubilization abilities, evidenced by clear solubilization halos on inorganic phosphorus agar and potassium solubilization media; (3) morphological observations using an optical microscope revealed it to be a Gram-positive bacterium; (4) growth curve analysis in liquid media with varying NaCl concentrations (0–15%) showed optimal growth at 5% salinity; and (5) biochemical profiling using Bacillus identification strips at 48 h, 72 h, and 96 h yielded positive results for Voges–Proskauer (V-*p*) reaction (+), citrate utilization (+), D-xylose metabolism (+), L-arabinose utilization (+), and nitrate reduction (+), while showing negative reactions for propionate utilization (−), D-mannitol metabolism (−), gelatin liquefaction (−), and starch hydrolysis (−). These findings collectively demonstrate the strain’s significant salt tolerance. The final isolated strain was identified as *Bacillus amyloliquefaciens.*

The species of foliar silicon fertilizer was Sodium metasilicate pentahydrate. The concentration of silicon fertilizer was maintained at 150 mg·L^−1^ according to the existing research results [87]. Foliar spraying and ST-PGPR were applied every 7 days when plants reached the two-leaf stage. A total of 7 treatments were used, each with 3 replicates, as detailed in Table 5.

### 4.3. Agronomic Management

A basal dose of compound fertilizer (N-P_2_O_5_-K_2_O = 15-15-15, 12 g·pot^−1^) was applied before sowing according to the local recommendations. Drip irrigation (flow rate: 2 L·h^−1^) was initiated when soil moisture, measured using a portable soil moisture meter, dropped below 75% of field capacity. Each pot received 500 mL of tap water. Seedlings were thinned to five uniform plants per pot at the two-leaf stage.

Throughout the trial, management practices (such as irrigation amount, irrigation method, agronomic measures, etc.) were maintained consistently.

### 4.4. Data Collection and Analysis

(1) Soil parameters: Soil moisture was measured using the gravimetric drying method. Electrical conductivity (EC, soil-to-water ratio 1:5) was assessed using a conductivity meter (DDB-303A).
(2) Crop antioxidant indicators:
  (1) Enzyme activities: The activities of catalase (CAT), superoxide dismutase (SOD), and peroxidase (POD), expressed in U·min^−1^g^−1^ FW (fresh weight), U·g^−1^ FW, and U·min^−1^g^−1^ FW, were measured by employing the UV absorption method, the nitrogen blue tetrazolium photochemical reduction method, and the guaiacol method at wavelengths of 240 nm, 560 nm, and 470 nm, respectively [88].
  (2) Osmolytes: The soluble protein content was determined using the Coomassie brilliant blue method at 595 nm [88], and the Proline content was measured using an ELISA kit (LabRe).
(3) Oxidative Damage: Malondialdehyde (MDA) content was quantified using the thiobarbituric acid (TBA) method at 452 nm, 532 nm, and 600 nm wavelengths [88].
(4) Phytohormone Quantification: The contents of Jasmonic Acid (JA), Salicylic Acid (SA), and Abscisic Acid (ABA) were determined using an ELISA kit (LabRe).

### 4.5. Statistical Analysis

Data were processed using Microsoft Excel 2019 (Microsoft Corporation, Redmond, WA, USA) and analyzed via SPSS 26.0 (International Business Machines Corporation, Armonk, NY, USA). A two-way ANOVA was performed to assess the main effects (ST-PGPR concentration, FSFS) and their interactions. Significant differences (*p* < 0.05) among treatments were determined using Duncan’s test. The principal component analysis (PCA) and figures were visualized using Origin 2024 (OriginLab Corporation, Northampton, MA, USA).

## 5. Conclusions

Based on a two-crop-cycle pot experiment, the regulatory influence of salt-tolerant plant-growth-promoting rhizobacteria (ST-PGPR) combined with foliar silicon fertilizer spraying (FSFS) on crop antioxidant performance was studied, and the results were obtained as follows.

The synergistic effects of ST-PGPR and silicon can significantly enhance the antioxidant defense mechanisms in plants under salt stress. In season 1, SOD activity increased by up to 2353.85% under the treatment of 10^4^ cfu·mL^−1^ ST-PGPR with foliar silicon. POD activity was consistently elevated by 19.79–153.29% in Season 1 and 3.44–118.31% in Season 2, with the highest values observed under the combined treatment of 10^4^ cfu·mL^−1^ ST-PGPR and FSFS. MDA content was consistently decreased by 8.12–35.87%, with a minimum observed at 10^4^ cfu·mL^−1^ ST-PGPR with foliar silicon in Season 1 and 10^6^ cfu·mL^−1^ ST-PGPR with foliar silicon in Season 2. The combined treatment of ST-PGPR and FSFS significantly increased accumulation of osmolytes such as proline and soluble protein. Proline content increased by up to 15.71%, with the maximal accumulation observed under the 10^8^ cfu·mL^−1^ ST-PGPR with foliar silicon in both growing seasons, while soluble protein content increased by up to 188.87%, with the maximal values achieved under 10^6^ cfu·mL^−1^ ST-PGPR with foliar silicon treatment in Season 1. Additionally, the levels of phytohormones, such as jasmonic acid (JA), salicylic acid (SA), and abscisic acid (ABA), were significantly regulated by the combined application of ST-PGPR and FSFS, further enhancing the plant’s stress tolerance mechanisms. JA content and ABA content increased by up to 31.81% and 30.09%, respectively, at 10^4^ cfu·mL^−1^ ST-PGPR in Season 2. SA content increased by up to 34.29%, with the highest observed at 10^8^ cfu·mL^−1^ ST-PGPR with foliar silicon in Season 2, demonstrating that the synergistic application of ST-PGPR and FSFS enhances defense-related hormone levels, thus strengthening plant resilience. This comprehensive evaluation indicated that SiM_4_ and SiM_6_ consistently ranked highest in terms of overall antioxidant performance across both seasons. This suggests that 10^4^–10^6^ cfu·mL^−1^ ST-PGPR combined with FSFS is particularly effective in enhancing antioxidant capacity and stress tolerance of Pak choi under salt stress.

Although silicon content in the leaves was not measured during the experiment in this study, and it is therefore impossible to confirm whether foliar silicon fertilizer was fully absorbed, the observed physiological improvements are consistent with previously established Si-mediated mechanisms. Future studies will address this limitation by analyzing the complete pathway of silicon uptake and distribution.

## Figures and Tables

**Table 1:** Soil moisture content (SMC) under ST-PGPR combined with FSFS.

Treatment	SMC/%			
	Season 1		Season 2	
	0–10 cm	10–20 cm	0–10 cm	10–20 cm
CK	11.67 ± 1.72 ab	10.99 ± 1.71 abc	14.15 ± 1.42 ab	12.94 ± 2.18 a
M_4_	12.20 ± 0.80 ab	11.09 ± 1.09 abc	14.49 ± 1.33 ab	14.14 ± 1.70 a
M_6_	15.43 ± 3.58 a	13.98 ± 2.67 ab	13.09 ± 0.70 b	12.25 ± 0.61 a
M_8_	11.96 ± 1.89 ab	11.06 ± 1.88 abc	13.70 ± 0.06 ab	13.25 ± 1.42 a
SiM_4_	10.07 ± 0.18 b	9.46 ± 0.42 c	15.00 ± 0.69 a	14.49 ± 0.52 a
SiM_6_	10.78 ± 0.79 b	9.84 ± 0.85 bc	14.50 ± 0.93 ab	14.42 ± 1.01 a
SiM_8_	15.92 ± 4.45 a	14.19 ± 4.37 a	12.98 ± 0.31 b	12.26 ± 0.94 a
ST-PGPR	ns	ns	ns	ns
FSFS	ns	ns	ns	ns
ST-PGPR × FSFS	*	*	ns	ns

Note: Data are presented as means ±  standard errors. Different lowercase letters in the same column indicate significant differences found with Duncan’s test (*p*  <  0.05). * represent a significant difference at the levels of 0.05, respectively; ns represents no significant difference. In 0–10 cm, ST-PGPR: F = 1.739, *p* = 0.065 in Season 1 and F = 2.723, *p* = 0.084 in Season 2; FSFS: F = 0.693, *p* = 0.419 in Season 1 and F = 0.903, *p* = 0.358 in Season 2; ST-PGPR × FSFS: F = 5.097, *p* = 0.022 in Season 1 and F = 2.143, *p* = 0.154 in Season 2. In 10–20 cm, ST-PGPR: F = 1.332, *p* = 0.304 in Season 1 and F = 1.456, *p* = 0.269 in Season 2; FSFS: F = 0.701, *p* = 0.417 in Season 1 and F = 0.669, *p* = 0.427 in Season 2; ST-PGPR × FSFS: F = 4.123, *p* = 0.039 in Season 1 and F = 2.149, *p* = 0.153 in Season 2.

**Table 2:** Electrical conductivity (EC) under ST-PGPR combined with FSFS.

Treatment	EC/(μS·cm^−1^)			
	Season 1		Season 2	
	0–10 cm	10–20 cm	0–10 cm	10–20 cm
CK	1462.00 ± 10.39 b	10.99 ± 1.71 abc	14.15 ± 1.42 ab	12.94 ± 2.18 a
M4	1403.00 ± 24.25 c	11.09 ± 1.09 abc	14.49 ± 1.33 ab	14.14 ± 1.70 a
M6	1058.00 ± 10.15 f	13.98 ± 2.67 ab	13.09 ± 0.70 b	12.25 ± 0.61 a
M8	1338.00 ± 11.53 d	11.06 ± 1.88 abc	13.70 ± 0.06 ab	13.25 ± 1.42 a
SiM4	1598.33 ± 6.51 a	9.46 ± 0.42 c	15.00 ± 0.69 a	14.49 ± 0.52 a
SiM6	1570.00 ± 48.88 a	9.84 ± 0.85 bc	14.50 ± 0.93 ab	14.42 ± 1.01 a
SiM8	1232.00 ± 20.42 e	14.19 ± 4.37 a	12.98 ± 0.31 b	12.26 ± 0.94 a
ST-PGPR	**	**	ns	ns
FSFS	**	**	ns	ns
ST-PGPR × FSFS	**	**	ns	ns

Note: Data are presented as means ±  standard errors. Different lowercase letters in the same column indicate significant differences found with Duncan’s test (*p*  <  0.05). ** represent a significant difference at the levels of 0.01, respectively; ns represents no significant difference. In 0–10 cm, ST-PGPR: F = 154.639, *p* < 0.001 in Season 1 and F = 0.748, *p* = 0.542 in Season 2; FSFS: F = 334.787, *p* < 0.001 in Season 1 and F = 0.026, *p* = 0.875 in Season 2; ST-PGPR × FSFS: F = 265.256, *p* < 0.001 in Season 1 and F = 0.026, *p* = 0.974 in Season 2. In 10–20 cm, ST-PGPR: F = 150.198, *p* < 0.001 in Season 1 and F = 1.022, *p* = 0413 in Season 2; FSFS: F = 167.817, *p* < 0.001 in Season 1 and F = 1.864, *p* = 0.194 in Season 2; ST-PGPR × FSFS: F = 64.046, *p* < 0.001 in Season 1 and F = 0.890, *p* = 0.433 in Season 2.

**Table 3:** Total variance explanation.

Growing Season	Principal Component	Eigenvalue	Contribution Rate (%)	Cumulative Contribution Rate (%)
Season 1	1	3.260	36.227	36.227
	2	2.243	24.925	61.152
	3	1.232	13.689	74.841
Season 2	1	4.904	54.490	54.490
	2	1.183	13.142	67.632
	3	1.029	11.432	79.065

**Table 4:** Comprehensive scores and ranking results of each treatment.

Growing Season	Treatment	Comprehensive Scores	Ranking	Growing Season	Treatment	Comprehensive Scores	Ranking
Season 1	CK	−1.473	7	Season 2	CK	−1.807	7
	M_4_	−0.070	5		M_4_	0.357	3
	M_6_	−0.127	6		M_6_	0.023	6
	M_8_	0.173	3		M_8_	0.083	4
	SiM_4_	1.003	1		SiM_4_	0.597	2
	SiM_6_	0.393	2		SiM_6_	0.723	1
	SiM_8_	0.100	4		SiM_8_	0.027	5

**Table 5:** Experimental treatments and design for salt-tolerant plant-growth-promoting rhizobacteria (ST-PGPR) and foliar silicon fertilizer spraying (FSFS).

Treatment	ST-PGPR Concentration	Silicon Fertilizer
CK	-	-
M_4_	10^4^ cfu·mL^−1^	-
M_6_	10^6^ cfu·mL^−1^	-
M_8_	10^8^ cfu·mL^−1^	-
SiM_4_	10^4^ cfu·mL^−1^	Sodium metasilicate pentahydrate
SiM_6_	10^6^ cfu·mL^−1^	Sodium metasilicate pentahydrate
SiM_8_	10^8^ cfu·mL^−1^	Sodium metasilicate pentahydrate

## Data Availability

The data used to support the findings of this study are available from the corresponding author upon request.
